# Triplet-triplet annihilation effects in rubrene/C_60_ OLEDs with electroluminescence turn-on breaking the thermodynamic limit

**DOI:** 10.1038/s41467-019-12597-5

**Published:** 2019-10-15

**Authors:** Xianfeng Qiao, Dongge Ma

**Affiliations:** Institute of Polymer Optoelectronic Materials and Devices, State Key Laboratory of Luminescent Materials and Devices, Center for Aggregation-Induced Emission, South China University of Technology, Guangzhou, 510640 P. R. China

**Keywords:** Organic LEDs, Photonic devices

**Arising from** S. Engmann et al. *Nature Communications* 10.1038/s41467-018-08075-z (2019)

Recently, Engmann et al. experimentally and theoretically examined the higher-order effects in rubrene/C_60_ organic light-emitting diodes (OLEDs)^1^, claiming that triplet-triplet annihilation (TTA) is not responsible for the electroluminescence (EL) with extremely-low turn-on voltage (V_oc_). Instead, the evidence from the equivalent circuit fitting suggest that the direct band-to-band recombination can interpret the low voltage phenomena. However, at applied bias of about 1.0 V, the excitons prefer to form on the charge-transfer state between rubrene/C_60_ interface. Direct singlet formation on rubrene and direct band-to-band recombination seems impossible to be accountable for such low V_oc_.

In heterojunction with direct band-to-band recombination, the EL intensity is determined by the chemical potential of the electron-hole pairs, as expressed by the generalized Kirchhoff and Planck equation derived by Würfel^2^. Assuming that voltage drop outside the heterojunction can be neglected, the chemical potential equal the external applied bias (V). The emitted photon current density per wavelength (*j*_*γ, λ*_) can be calculated as a function of voltage using Eq. (1). This can be further transformed into EL intensity (L) by taking luminous efficiency function into consideration in visible range (Eq. (2)).

1 $$dj_{\gamma ,\lambda } = \frac{{2a\left( \lambda \right){\mathrm{\Omega }}c}}{{\lambda ^4\left( {{\mathrm{exp}}^{\frac{{h{\mathrm{c}}/\lambda - eV}}{{kT}}} - 1} \right)}}d\lambda$$

2 $$dL = k_mv\left( \lambda \right){\mathrm{E}}_{ph}dj_{\gamma ,\lambda }/{\mathrm{\Omega }} = \frac{{2k_mv\left( \lambda \right)a\left( \lambda \right)hc^2}}{{\lambda ^5\left( {{\mathrm{exp}}^{\frac{{h{\mathrm{c}}/\lambda - eV}}{{kT}}} - 1} \right)}}d\lambda$$

where *a*(*λ*) is the absorptance, Ω is the solid angle and equals π for flat device, *k*_*m*_ = 683 lm/W is the maximum luminous efficiency at wavelength 555 nm, *v*(*λ*) is the luminous efficiency function, E_*ph*_ = *hc*/*λ* is the photon energy, c is the light velocity, *h* is the Planck constant, *k* is the Boltzmann constant and T is the temperature. Here, assuming an absorptance of 1 and that the absorption spectrum is a rectangle of 50 nm width, the absolute photon current density and EL luminance can be approximately calculated for estimating the thermodynamic limit of EL turn-on^3^.

**Fig. 1:**
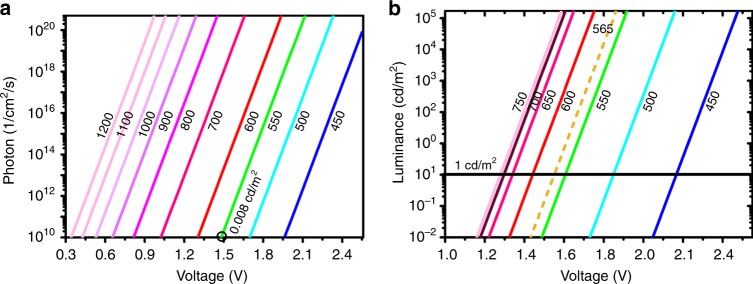
Calculated emission intensity. The photon current density (**a**) and luminance (**b**) as a function of voltage for various color OLEDs. 10^10^ photon/cm^2^/s @550 nm corresponds to 0.008 cd/m^2^. The short-dashed line in **b** is for rubrene emission with peak of 565 nm. The black line is guide for eyes of 1 cd/m^2^

Clearly in Fig. 1a, V_oc_ (@10^10^ photon/cm^2^/s) is highly emission color-dependent. This well interprets the common low turn-on phenomenon in inorganic LEDs^4^ that 870 nm emission can be observed at about 1.2 V. Similarly, the charge-transfer emissions with peak ranging from 800 to 1200 nm in organic photovoltaics^5^ can be detected below 1V with direct band-to-band recombination mechanism. Figure 1b displays the EL luminance vs voltage in visible range. As the EL peaks shift from 450 to 750 nm, V_oc_ (@1 cd/m^2^) reduces from 2.17 to 1.28V. Another feature is that the theoretical V_oc_ is much lower that the optical bandgap. As in rubrene with 2.2 eV optical bandgap, the thermodynamic limit is only about 1.55 V. Adding the voltage drop outside the emissive layer, the actual V_oc_ of 2.0 V is found in device with structure of ITO/PEDOT:PSS/rubrene/BCP/Al^1,6^. In short, the calculation value is important indictor for judging whether it is necessary to invoke a new mechanism to replace the direct recombination mechanism^7,8^.

Experimentally, the rubrene/C_60_ devices exhibit an extremely low V_oc_ of about 1 V^1,6,9^, breaking the thermodynamic limit stated in direct recombination mechanism (Fig. 1b). The 0.9 V turn-on is coinciding with the charge-transfer emission peaking at 890 nm in rubrene/C_60_ OLEDs^10^. Specifically, the excitons forming on the charge-transfer state can transfer to the rubrene triplet states, followed by the TTA process^11–14^. The TTA mechanism enables the observation of EL below the theoretical V_oc_ derived from the direct band-to-band recombination mechanism. It has been reported that TTA can take place in non-coherent sunlight level (2 × 10^14^ photons/cm^2^/s/nm@532 nm)^15^, and hence also works in the operating condition of practical OLEDs (>1 mA/cm^−2^ = 6.25 × 10^15^ electron/cm^2^/s). Previous reports with various evidences^11–14^ also support that TTA mechanism is necessary for the anomalous low turn-on phenomenon in rubrene/C_60_ devices.

Engmann and coworkers rules out the contributions of TTA around turn-on voltage mainly based on the ideality factor value from current-voltage modeling. The valid of diode equation for organic heterojunction is debatable and needed more thorough consideration. The calculation of the V_oc_ can be used to determine whether a new mechanism is involved or not.
